# Modified Falling Mass Impact Test Performance on Functionally Graded Two Stage Aggregate Fibrous Concrete

**DOI:** 10.3390/ma14195833

**Published:** 2021-10-06

**Authors:** Nandhu Prasad, Gunasekaran Murali, Nikolai Vatin

**Affiliations:** 1 School of Civil Engineering, SASTRA Deemed to be University, Thanjavur 613401, India; nandhuprasad@sastra.ac.in; 2 Peter the Great St. Petersburg Polytechnic University, 195251 St. Petersburg, Russia; vatin@mail.ru

**Keywords:** modified impact test, steel fibre, polypropylene fibre, knife-edge specimen, coefficient of variance, multiphysics model

## Abstract

This research examined the performance of functionally graded two-stage fibrous concrete (FTSFC) against modified repeated falling-mass impacts. This study led to the concept of creating improved multiphysics model of fibre composites with better impact resistance for potential protective constructions. FTSFC was developed based on the bio-inspiring strength of turtle shells. The excellent impact resistance of FTSFC was accomplished by including a larger quantity of steel and polypropylene fibres in the outer layers. At the same time, one- and two-layered concrete were cast and compared to evaluate the efficiency of three-layered FTSFC. To minimize the dispersed test results, a modified form of the 544 drop-mass impact test was recommended by the American Concrete Institute (ACI). The modification was a knife-edge notched specimen instead of a solid cylindrical specimen without a notch. This modification predefined a crack path and reduced the dispersion of results. Cracking and failure impact numbers, ductility index, and failure mode were the testing criteria. The suggested modification to the ACI impact test decreased the coefficient of variance, showing that the dispersion of test results was reduced significantly. This study led to the concept of creating improved, fibre composites with better impact resistance for potential protective constructions.

## 1. Introduction

**Figure 1:**
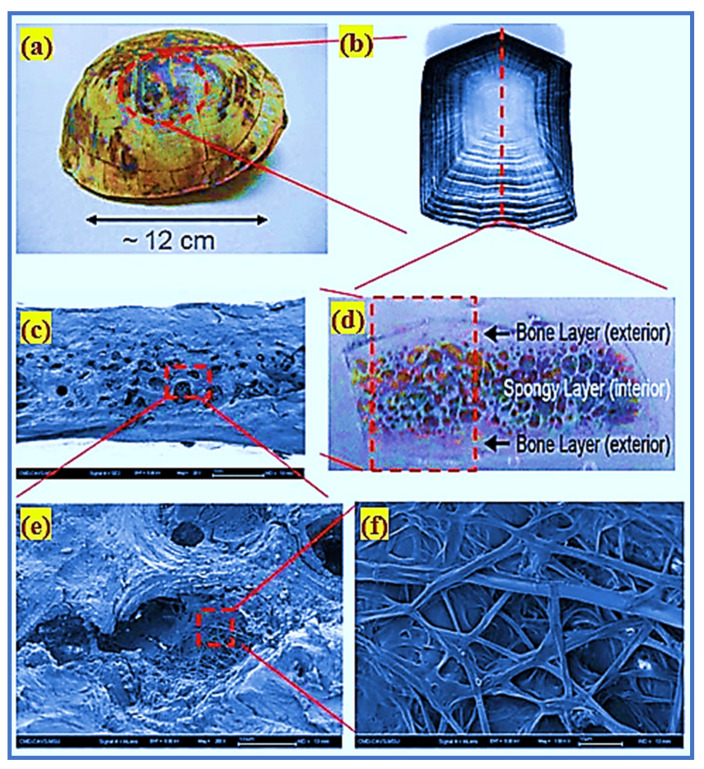
The structure of turtle shell and its multiscale hierarchy: (**a**) a morphology of the shell structure of turtle; (**b**) a costal scute indicating the sequent pattern of growth pattern; (**c**) scanning electron microscope image of the fractured surface; (**d**) a cross-section of carapace displaying a composite layer; (**e**) scanning electron microscope image of the cell structure; and (**f**) scanning electron microscope image of the interior fibrous structure of the cell.

The construction industry is developing rapidly due to novel composite materials and methods. One of the most emergent methods is the bionic-inspired design of functionally graded composites. These biomaterials have recently earned attention from researchers owing to their novel hierarchical structures and high impact strength [1]. Exploring a biomimetic structure or material enables researchers to invent new and creative design concepts. The idea of utilizing biomaterials is a novel insight into material technology, and this study area is referred to as biomimicry in a specific group. Composite concrete materials could provide a creative biological mix and more significant impact-resistant characteristics (e.g., using the carapace of turtle and tortoise) [2]. Nano/micro/mesoscale-level complex structures are composed of organic and inorganic components that are spatially structured. They are protected from impact stress and also guarantee flexible movement by the turtle shell. Figure 1 shows the sandwich type of the turtle’s carapace shell. The turtle shell has three layers; the first layer is very dense and shielding and is termed endocortic; the second layer is porous, and acts as an impact absorber. The third layer is extremely thick and exotic, and provides shielding [3]. Turtle shell exhibits superior armor properties and serves as a model for using the endocortical layer to resist piercing and the trabecular layer to absorb the applied load. Differences in their composition and structural systems are due to the remarkable strength properties of biomaterials.

Construction materials such as concrete are the most commonly used in the building sector due to the growing civilian and military infrastructure needs. A drastic increase in living standards, combined with a sharp rise in the global population, can be attributed to this development [4]. The current infrastructures, tall buildings, long-span bridges, and transportation structures should be enhanced to resist accidental loads. As a result, the properties of concrete such as impact resistance, energy absorption, and durability will be improved to withstand a wide range of loading conditions. Concrete is subjected to high repeated impact loads due to its use in barriers, ad protective structures [5], and other applications. When the structural components are subjected to impact loads, they can suffer significant damage to their structural stability and integrity. When structural damage occurs, the remaining strength of the structure will be doubtful. As a result, the use of impact-resistant concrete in designs is recommended to maintain the impact loading effectively. Several researchers have used different materials to enhance the concrete strength, such as chemically depolymerized waste polyethylene terephthalate (PET) aggregates [6], fibre-reinforced polymer (FRP) confinement [7], carbon-fibre-reinforced polymer (CFRP) jacketing [8], ceramic-ball aggregate fibrous geopolymer composite [9], and fibrous concrete [10].

### 1.1. Evolution of Fibrous Concrete

Fibre-reinforced concrete (FRC) is a frequently used composite material in the construction sector, since it increases the efficiency of concrete buildings subjected to various impact loadings. FRC is a promising material for modernizing various impact-resistant applications in critical structures, vehicle crashes on transportation structures, flooring, a long-haul viaduct, military facilities, radioactive storage, and airstrip pavements. The most vulnerable buildings are expected to be unaffected by a single powerful explosion, but structures subjected to repeated collisions exhibit damage, resulting in collapse. As a result of increasing artificial hazards, there has been a rising need to enhance the structural integrity of infrastructure exposed to repeated impacts and explosions. On this basis, several researchers have been employed to improve the FRC materials, which can exhibit a superior impact resistance to conventional concrete. The development of material technology and unification has enhanced the emergence of a new named composite: two-stage fibrous concrete (TSFC) [11].

TSFC is a unique type of concrete that is manufactured using a different casting technique than traditional concrete. Many distinct names throughout the world are used for two-stage concrete: Colcrete, Polcrete, Arbeton, Naturbeton, prepacked concrete, grouted aggregate concrete, and injected aggregate concrete [12,13,14,15]. In TSFC, the coarse aggregate volume and fibres are packed in the formwork and interlocked [16] Subsequently, spaces between the aggregates and fibres are filled with a flowable grout to complete the casting [17]. Increasing the coarse aggregate content in TSFC alters the concrete’s characteristics due to inherent interlocking—increased aggregate contact points leading to improve stress distribution under loading [18,19]. Two-stage concrete has been widely utilized in various applications, including high-density coarse aggregates for radiation shielding, submerged structures, mass concrete structures, and tunnels [20]. Several investigations have been performed to examine the mechanical performance of TSFC [21,22,23]. Nehdi et al. (2017) [24] investigated the mechanical characteristics of TSFC made with various percentages (1%, 2%, 4%, and 6%) of long and short steel fibres. The results revealed that increasing the fibre dosage could significantly enhance the tensile and compressive strength. Moreover, the inclusion of 6% steel fibre enhanced the flexural toughness, flexural strength, and concrete performance. A study indicated that the sand-to-cement and water-to-cement ratios, porosity, and the strength of the cement grout and coarse aggregate also mainly influenced the two-stage concrete’s overall compressive strength [25]. Murali et al. (2019) [18] investigated the impact response of TSFC comprising long hooked-end and crimped fibre at percentages (1.5, 3, and 5%) by volume. Findings indicated that the two-stage concrete that contained a 5% dose of long hooked-end fibres showed better impact resistance and compression strength. Murali and Ramprasad (2018) [26] examined the effect of layered TSFC slabs against mass free-fall impact. Three-layered TSFC slabs, each with a distinct fibre dosage of 4%, 2%, and 4%, were used for the top, mid, and bottom layers, respectively. The findings demonstrated an excellent impact energy absorption capability in layered TSFC, eliminating breakability and delaying crack development and expansion [27]. Numerous studies were conducted to enhance the potential application of this type of concrete in the construction sector. Recently, the development of TSFC has moved toward functionally graded fibrous concrete (FGFC).

### 1.2. Functionally Graded Concrete (FGC)

The advancement of material technology and their unification has resulted in novel composites known as FGC. Fibrous FGC is a novel concrete composite with enhanced mechanical characteristics that ensures the required behavior by altering its properties. Greater density with a stronger fibre matrix is possible in functionally graded fibrous concrete (FGFC), which has outstanding toughness, uniaxial tensile strength, and impact resistance [28,29]. Moghadam and Omidinasab et al. [30] researched the impact behavior of FGFC slabs containing a hybrid combination of 1% nylon fibre and 1% steel fibre. The results showed that the increase in flexural strength of FGFC in the nylon fibre was smaller than in the steel fibre. When hybrid, steel, and nylon fibres were introduced, there was a significant increase in flexural strength, by about 1.7, 2.6, and 1.2 times, respectively. When FGFC was compared to ordinary fibrous concrete, it demonstrated a substantial increase in flexural strength. Sridhar and Ravi Prasad et al. [31] investigated three-layered FGFC beams with designed cementitious composites containing 1.5 and 0.5% polyvinyl alcohol and steel fibre, respectively, throughout the 25 mm-thick stress zone. The results showed that the load-carrying capability of the multilayered FGFC beams increased up to 36%. Nandhu Prasad and Murali [11] investigated the effect of three-layered FGFC cylindrical specimens when subjected to drop-mass impact. FGFC specimens with varying steel and polypropylene fibre doses in each layer were provided in two- and three-layered configurations. Findings revealed that the three-layered FGFC specimens with a steel fibre content of 0% in the middle layers and 3.6% percent in the top and bottom layers were found to have the highest impact resistance. The failure impact strength of multilayered specimens compared with single-layered specimens with the same quantity of steel fibres rose by 39%. It was found that using the idea of layered fibre composites with a greater dosage of fibres in the two layers resulted in outstanding fibre efficiency and better impact resistance. Regarding examples of the creation of FGFC using two-stage concrete, there is only a small amount of research accessible in the literature. In order to fully understand the energy-absorption capability of this material, as well as its impact performance, further study is needed.

### 1.3. ACI 544 Drop-Mass Impact Test

Several test techniques have been described in the literature [32] for evaluating the impact resistance of FRC; for example, drop mass [33,34], projectile [35,36,37], blast [38,39], and Charpy pendulum [40,41,42]. Nonetheless, a few of these mentioned tests are costly and difficult to conduct. The ACI 544 [32] standard established a simpler method for qualitatively evaluating the impact resistance of FRC using a drop-mass test. An impact force is repeatedly applied to a cylindrical specimen until it cracks and fractures. The ACI 544 method of testing demonstrated a large dispersion in experimental results due to the following factors [16,17,18]: (1) scattered test findings as a consequence of visual monitoring identifying the crack start and final crack, which may occur in any direction; (2) impact strength values are derived from a single impact point on the concrete’s surface that occurs on the cement matrix with a soft layer and coarse aggregate with tough solid surface; (3) concrete is a nonhomogeneous material, resulting in variations in the design of mixtures, which leads to dispersion of impact strength results; and (4) this test must involve physical phenomena—pulling up and dropping a mass of 4.54 kg from a specified height for a specific duration. In view of these factors, the researchers concluded that a statistical technique was the best method for examining the dispersed experimental impact-test findings.

**Figure 2:**
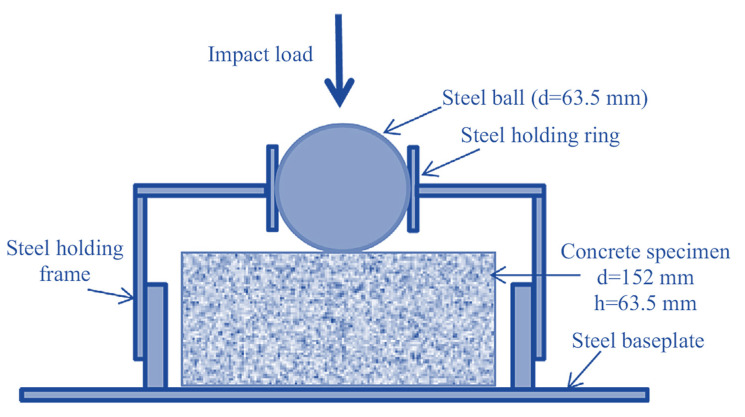
The ACI drop-weight impact test.

Several researchers have recently utilized the ACI 544 repetitive falling drop weight or mass impact (RFDWI) test to assess the impact resistance of fibrous cementitious materials [17,18,43,44,45,46,47,48,49,50,51,52,53]. The main issue with the RFDWI test is the large dispersion in results [54]. Several studies have examined the statistical variance of RFDWI test results for different fibrous concretes [55,56,57,58,59,60]; these findings were not regularly distributed [49,54,55,56,57,58,59,60,61,62,63]. Numerous researchers have calculated the coefficient of variance (COV) values and the ‘t’ value of the student’s t-test to determine the minimal number of test specimens needed to maintain the error within a given range [54,55,58,60,61,62,63,64,65,66,67]. Several researchers [17,18,45,53,65] used Weibull distribution to examine the dispersed impact findings. Nataraja et al. [55] examined the statistical fluctuation of RFDWI test results for steel FRC; the results indicated COV values of 57.3 and 46.5% for the first crack and failure for the first and second batches, respectively. Badr et al. [58] reported that each test required at least 40 test specimens to maintain the error below 10%. The calculated COVs were 56.6 to 61.4% for the first crack and 48.7 to 52.1% for the failure of two batches of 20 specimens each. Rahmani et al. [59] reported RFDWI test findings for carbon, polypropylene, and steel FRC specimens that showed a greater COV of polypropylene FRC, ranging from 51 to 62% compared to other fibrous concrete. Murali et al. [60] examined the reliability of RFDWI test findings for steel FRC. Modifications in mechanical characteristics of FRC also must be addressed when determining the minimum number of tests required to assess impact strength, and the COV found in investigational impact-test results typically surpasses 40%. Accordingly, many specimens are needed to guarantee advancement to favorable conclusions about the normal distribution of test findings [55]. Various testing methods were performed to assess the resistance of FRC against impact, and the ACI-suggested drop-weight test was the easiest and most attractive. The ACI drop-weight test involves the free fall of a 4.54 kg steel ball from an elevation of 457 mm onto the specimen’s top surface. The radius and thickness of the cylindrical specimen were 76 and 64 mm, respectively. Figure 2 illustrates the strong steel plate that supported the specimen, four positioning lugs that restricted movement in the lateral direction, and a positioning bracket that secured a 63.5 mm steel ball placed on the specimen that acted as a load distributor. This test produced only recorded impact numbers corresponding to cracking and failure of the test specimen.

Consequently, this test made it simple to determine the relative energy engrossment of various materials. The pilot investigations of the drop-weight impact testing on different kinds of concrete are shown in Table 1. The COVs in Table 1 show that the ACI impact test results were highly dispersed, which could be attributed to the following:

- A single-point impact on the specimen increased the chance of inaccurate findings—the point of impact could be a soft cement matrix or a hard coarse aggregate;
- Cracks were permitted in any direction and anywhere on the specimens—this strengthened the test subjectivity when cracking was inspected visually;
- Due to the lack of a standard, the test findings were dispersed;
- The specimen base, as well as the apparatus lug, were characterized as failures, and even with excessive fracture width, this failure observation could produce a recurrent impact on the specimen;
- Preparation of specimens was not based on any standard, so the specimens may have had mold-faced surfaces.
- Despite the numerous benefits of the ACI drop-weight impact test, the dispersed results were the primary disadvantage that must be addressed.

### 1.4. Proposed Modification to the ACI 544 Test Method

In this study, the leading two causes of inaccuracy were examined, and changes were proposed accordingly. Badr and Asour et al. [54] identified five factors that contributed to the large variations of the ACI 544 test findings [69]: (i) the various acceptable preparation methods of specimens might be regarded as another source of variance. ACI 544 specifies that disc specimens may be cast as per the specified dimensions or cut from the standardized cylinders for the compressive strength of the concrete; (ii) the criteria for considering the specimen’s failure may result in the impact being halted before failure or continuing after failure, resulting in erroneous records of the final number of blows. Additionally, there were no particular suggestions about failure mechanisms that should be avoided; and (iii) two potential sources of the dispersion of results were imposed by the load arrangement of an axially applied impact force on the centre steel ball:

- It permitted the formation of cracks in any direction, complicating the identification of the initial visual crack;
- Since concrete has a heterogeneous material feature, the centre point that was immediately exposed to the focused force could be a coarse solid aggregate grain or a soft mortar region. As a consequence, results that will not accurately reflect the material’s impact strength could be achieved.

**Figure 3:**
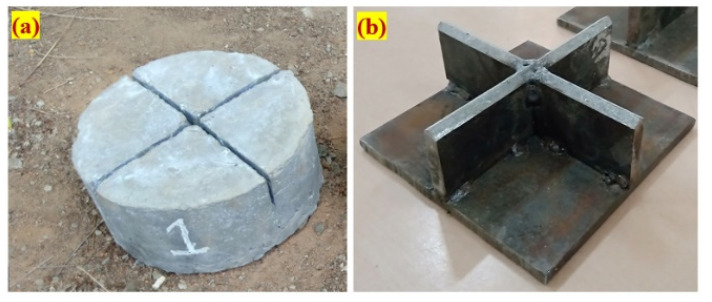
Details of the proposed modification: (**a**) notched specimen; (**b**) cross knifelike plate.

Figure 3 depicts the proposed cross notch on the specimen’s top surface and a cross knifelike load-transmitting plate. When the hammer was repeatedly dropped against the load-transferring plate, the impact load was dispersed across a larger area instead of concentrated in a single location. Secondly, due to this alteration, fractures could develop parallel to the path of contact, resulting in the specimens splitting into four parts, without any numerous cracks forming in a radial pattern. This alteration predefined the cracked route and failure of the specimens, leading to significantly reduced dispersion of results.

## 2. Research Significance

There is a shortage of adequate information in the bibliography regarding modified testing procedures for the ACI drop-weight test. Despite this, researchers worldwide have utilized different statistical methods to evaluate the substantial dispersion in ACI drop-weight test data. It is fascinating to perform the modified impact test and reach a logical conclusion to minimize dispersion outcomes. However, very little study has been conducted to decrease dispersion outcomes by adding granular bedding, and gaps in this research field are still open. To fill these research gaps, this study sought to evaluate the impact performance of FTSFC by performing a modified impact test. A simple technique was suggested to minimize dispersion effects by utilizing a notched specimen and a cross knife plate instead of a non-notched specimen and a steel ball. The impact number related to cracking and failure, failure mechanism, and ductility index were studied in detail in our research.

## 3. Experimental Investigation

### 3.1. Base Materials

**Figure 4:**
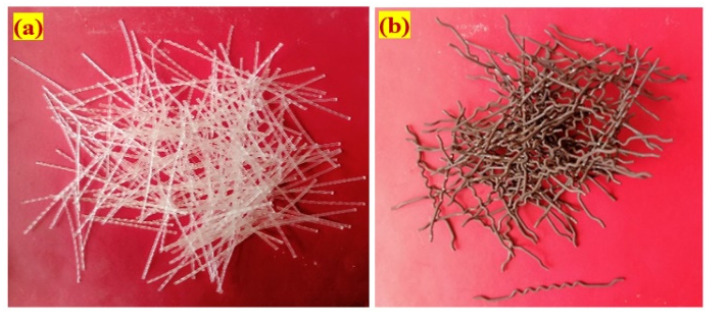
The appearances of each fibre (**a**) Polypropylene (**b**) Steel.

- The cement used in this research had a specific gravity of 3.14 as per the IS 1489-2015 standard [70]. The conventional consistency of the blain fineness was 375 m^2^/kg; the consistency was 30.8%; and the initial and final setting times were 32 and 550 min, respectively.
- Natural river sand was used as the fine aggregate, meeting the requirement of IS 383-2016 [71]; the gradation curve was consistent with Zone II; the specific gravity was 2.65; and the fineness modulus was 2.41. The fine aggregate particle size was less than 2.36 mm, resulting in an excellent flowable grout blend in accordance with ASTM C939/C939M-16a [72].
- The coarser aggregate utilized were natural gravel with a size of 12.5 mm meets the requirement according to IS 383-2016 [71]. The apparent bulk density of the coarse aggregate was 1700 kg/m^3^, the specific gravity was 2.6, and the water absorption percentage was 0.56.
- The commercial superplasticizer Tech Mix 640 was utilised to reduce water and extend grout time in the plastic stage. A grout fluidifier typically is composed of a water-reducing additive at a suggested dose of 1% by cement content [73]. The water-reducing admixture dose was restricted to 0.4% in this research to provide excellent efflux time and flowability, and to avoid honeycombing.
- Fibre is widely used as a component in concrete reinforcement due to the many advantages it provides in this application. A new geometrically shaped macro polypropylene fibre (PF) and steel fibre (SF) were utilized in this study; the unique PF was 45 mm in length and 0.8 mm in diameter, with a tensile strength of 500 MPa; and the hybrid hooked-end crimped SF had a length of 50 mm, a diameter of 1.0 mm, and a tensile strength of 1200 MPa. The appearances of the PF and SF used in this research are illustrated in Figure 4.

### 3.2. Mixing Composition

Twelve different concrete mixes were developed in the present study to assess their impact resistances. A series of grout blends were developed to select the optimal grout based on the cone-test efflux time, which met the compression strength and flowing grouting criteria. The optimized grout efflux time ranged from 35 to 40 s, as per the American Society for Testing and Materials (ASTM) C939 standard [72]. The optimum ratios were 1.0 and 0.45 for cement to sand (c/s) and water to cement (w/c). In order to enhance grout flowability, a high-range water reducer was introduced to the water. Its dose for nonfibrous specimens was 0.3%, and for fibrous specimens was 0.5%. Table 2 shows the composition of the 12 blends with various fibres and doses in each layer of the FTSFC. The first combination was deemed a fibre-free reference specimen and labeled as PAC. The second and third mixtures were fibrous composites with SF and PF in a single layer. The first letter ‘S’ indicated a single layer of concrete, while SF or PF indicated the kind of fibre utilized. The fourth and fifth mixtures were FTSFC with a double layer and were respectively labeled as D-SF-PF and D-PF-SF. The first letter indicated a double-layer FTSFC for that group, while SF-PF or PF-SF indicated the type of fibre in the top and bottom layers, respectively. The final seven mixtures were three-layered FTSFCs, with various 2.4% dose fibre schemes, which were labeled as T-FG1 to T-FG7.

### 3.3. Method for Preparation of a Specimen

**Figure 5:**
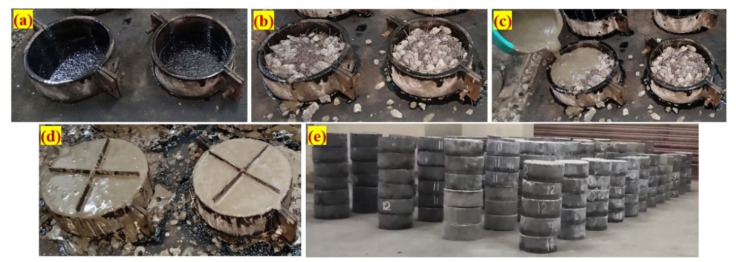
The fabrication method: (**a**) empty cylindrical mold; (**b**) fibres and coarse aggregate filled into the mold; (**c**) pouring of grout; (**d**) casted specimens with notch plate insertion; and (**e**) appearance of specimens, which were kept on the floor after demolding.

A total of 180 cylindrical specimens were produced, with 15 per mixture. To assess the impact strength, cylindrical specimens with a diameter of 152 mm and a height of 64 mm were utilized. The FTSFC step-by-step casting technique involved three essential steps, as shown in Figure 5. The empty cylinder mold was kept on a level surface and oil was used to coat the entire interior, as shown in Figure 5a. Second, aggregates and fibres were initiated and packaged in an empty mold to create a natural skeleton, as shown in Figure 5b. Third, the cement grout was poured over the top of the created skeleton, which enabled the gaps to be filled by gravity in the skeleton, as illustrated in Figure 5c. A small compact was applied to ensure that the grout filled all voids. The appearance of specimens after grouting is shown in Figure 5d. After 24 h, all specimens were removed and their appearances sorted in order, as shown in Figure 5e. The specimens taken from the mold were cured and evaluated for 28 days.

### 3.4. Test Setup for Drop-Mass Impact

**Figure 6:**
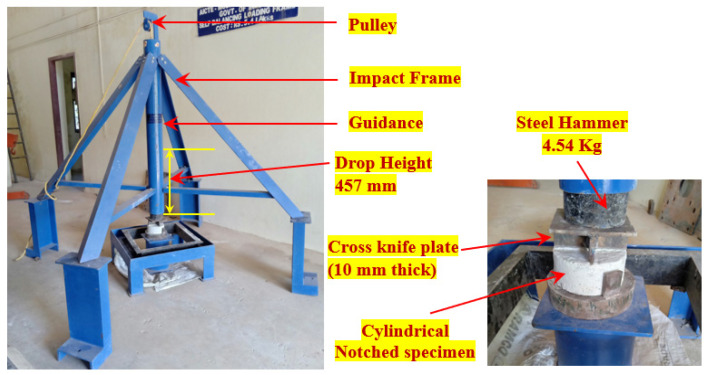
The Impact-test setup.

In accordance with ACI Committee 544–2R, a modified drop-mass impact test was performed to assess the impact strength of the FTSFC specimens [32]. The test technique and method for the modified drop-weight impact test were straightforward when displacement, time history, and vibration were not measured. In the test a 4.45 kg steel hammer was raised and dropped freely from a height of 457 mm onto the top of the cross knifelike plate positioned on the top of the notched specimen. Lateral movements of the specimen were avoided upon impact by keeping the specimen on a four-legged steel plate. The modified drop-weight testing equipment utilized in this study is shown in Figure 6. Visual inspection documented the cracking and failure impact numbers of the specimen. When a crack reached the specimen on the bottom, it was defined as a failure and separated into two parts.

## 4. Discussion of Results

### 4.1. Compressive Strength of FTSFC

**Figure 7:**
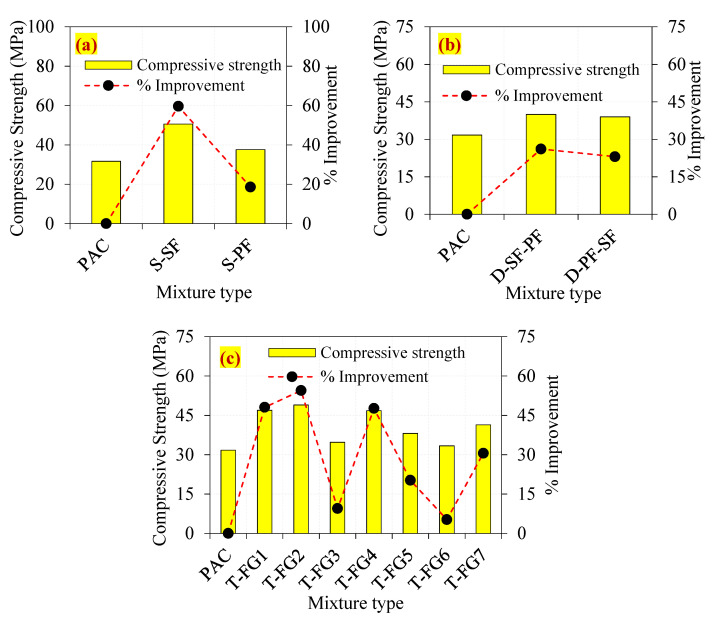
The observed compressive strengths of the FTSFC (**a**) Single layer (**b**) Double layer (**c**) Triple layer.

For each type of combination, three cubical specimens were cast and tested for compressive strength in accordance with the standards of IS 516 [74]. The compressive strength of the single-layer preplaced aggregate fibre concrete containing SF and PF is shown in Figure 7a. When compared to the PAC specimen, the S-SF specimen had a compressive strength that was 59.6% higher. This enhancement was related to the existence of SF, which formed effective bridges inside the cracking zone, delaying the start and progression of cracks [75]. In comparison to PAC, the compressive strength of the S-PF specimen increased by just 18.6%. The compressive strength of PF was lower than the compressive strength of the SF. This was attributed to PF having a lower tensile strength and density than SF [2,11]. As shown in Figure 7b, the double-layer D-SF-PF and D-PF-SF specimens demonstrated a 26.1 and 23.0% improvement in compressive strength, respectively, compared to the PAC. The use of SF in single- and double-layer concrete, according to the findings, significantly improved the compressive strength of the concrete. The results indicated that adding SF to both single- and double-layered concrete increased the compressive strength considerably. Moreover, single-layered concrete outperformed double-layered concrete. This was justified by the homogenous distribution of 3D-oriented fibres in the composite, which increased its load-carrying capability under compression.

Compared to PAC, three-layered FTSFC reinforced with various fibre doses showed an improvement in compressive strength that varied from 5% to 54%. The lowest compressive strength found in this category was for the T-FG6 mix, which had a compressive strength that was 5.2% greater than the PAC mix, as shown in Figure 7c. The top and bottom layers were 3.6% PF with poor tensile strength, while the intermediate layer was composed of nonfibrous concrete. Compared to PAC, the T-FG2 specimen, with 2.8% SF in the top and base layers and 1.6% in the intermediate layer, demonstrated increased compressive strength up to 54.4%. All other three-layer FTSFC specimens showed the desired increase in compressive strength. The addition of more mono- and hybrid fibres to the different schemes resulted in substantial compressive strength improvements. The incorporation of fibres into concrete increased its bridging ability significantly. The crack path was intricate, requiring tremendous effort to remove the fibre activity [2,11]. In particular, the fibre content of ordinary fibrous concrete was restricted to 2% due to workability issues, uniform fibre distribution, and fibre clustering, all of which contribute to increased voids, resulting in internal concrete flaws and a decrease in compressive strength [12,24]. In comparison, the FTSFC casting technique eliminated these problems by premixing and preplacing the coarse aggregate and fibres in the mold before grout injection [11]. In conclusion, the effect of SF on compressive strength was considerably higher than the effect of PF for different concrete layers.

### 4.2. Impact Test Results

This study investigated the impact behavior of FTSFC specimens under falling mass impact. The impact numbers causing the initial crack (Q1) and the final crack (Q2) are listed in Table 3 for 12 distinct mixes. The average values of 15 specimens were used for the discussions.

#### 4.2.1. Effects of Single-Layered Concrete

**Figure 8:**
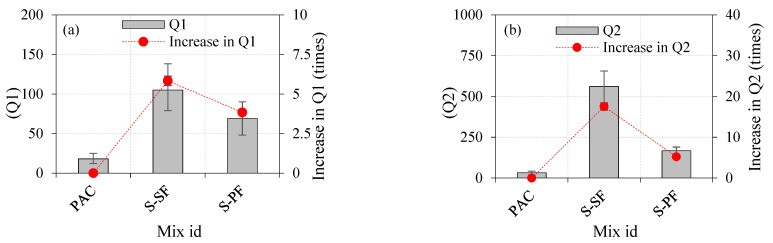
The effects of a single-layered specimen on the impact strength (**a**) Q1 (**b**) Q2.

The effects of a single-layered specimen on the impact strength are shown in Table 3. Fibre addition in concrete generally improved the impact-resistance performance. As shown in Figure 8a,b, the Q1 and Q2 values of the control concrete (PAC) were 18 and 32, respectively. The Q1 and Q2 values for the S-SF specimen were 104 and 563, respectively. The observed values were increased by 5.83 and 17.53 times, respectively, compared to the PAC specimen. The values noted for Q1 and Q2 in the S-PF specimen were 67 and 162, respectively. The observed values increased by 3.83 and 5.21 times, respectively, as compared to the PAC specimen. Compared with the S-PF specimen, the Q1 and Q2 for the S-SF specimen were increased by 55% and 248%, respectively.

The effect of fibre addition and its distribution in concrete was the most common cause of a rise in Q1 and Q2. Uniformly dispersed fibres inhibited micro/macrofracture development, allowing for improved stress homogeneity in the concrete by distributing stress concentration over a larger region. The fibre bridging effect was accounted for by changing the crack direction, intensifying the crack progression, and minimizing the crack width [39]. Cracks began to appear on the micro- to macroscale in the beginning. By interconnecting both micro- and macrocracks in the stress area, PF and SF fibres prevented micro- and macrocracks, respectively. This promoted stress transmission across the fractured region, thus improving the concrete’s residual strength [76,77]. When evenly distributed in the matrix, each fibre served as a marginal impact-energy-absorption element, sharing a certain load upon impact. SF improved the concrete’s capacity to prevent macrocrack propagation during impact loading and efficiently transfer tensile loads after crack formation. Additionally, owing to the frictional interaction between the matrix and the fibres, PF insertion bridged microcracks and retarded their growth [78]. The rise in Q1 and Q2 for the S-SF and S-PF specimens compared to PAC was due to a greater fibre dosage (2.5%) being evenly distributed across the section, which resulted in a significant decrease in the concrete’s brittleness.

#### 4.2.2. Effects of Double-Layered FTSFC

**Figure 9:**
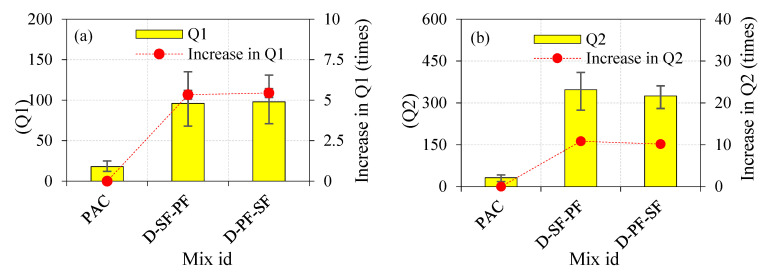
The effects of the double-layered FTSFC on impact strength (**a**) Q1 (**b**) Q2.

Figure 9a,b illustrates the Q1 and Q2 of the two-layered FTSFC. We recorded a tendency toward increase in Q1 and Q2 compared to the PAC specimens. The Q1 and Q2 values for the D-SF-PF specimen were 96 and 347, respectively. Compared to the PAC specimen, the recorded Q1 and Q2 values increased by 5.33 and 10.84 times, respectively. Likewise, the D-PF-SF specimens exhibited Q1 and Q2 values of 98 and 325, respectively. The recorded Q1 and Q2 values increased by approximately 5.44 and 10.15 times, respectively, compared to the PAC specimen. This clearly showed that the two-layered FTSFC that comprised SF and PF in the top and bottom layers, and vice versa, exhibited a higher impact resistance and ductility, and eliminated early brittle failure [79]. A 2.4% dosage of SF in the top layer of the two-layered FTSFC had a critical role in altering the cracking mechanism in D-SF-PF compared to D-PF-SF. This phenomenon was due to the presence of SF at the point of impact, which led to more energy being absorbed in the top layer. Adding SF improved the bridging effect with the surrounding matrix, resulting in increased strength and resistance to fibre pull-out from the matrix, and enhanced the behaviour of crack restraint and effective tensile stress transfer along with the fractured segments of FTSFC’s top layer [80]. On the other side, it provided PF in the top layers, resulting in the cracking that occurred rapidly and propagated to the bottom layer with SF. The effectiveness of impact resistance of the D-PF-SF was less than that of the D-SF-PF specimen. When comparing the D-PF-SF and D-SF-PF specimens, an SF top layer exhibited better performance due to fibre-bridging action in the cracked region, which delayed crack opening and development [75].

The crack propagation in the D-PF-SF specimen was slow when compared to the D-SF-PF specimen. The D-PF-SF specimen contained a 2.4% dosage of PF in the top layer and SF in the bottom layer. The fracture began on the top surface and rapidly expanded into the second layer under falling-mass impact. The second layer resisted the crack propagation more effectively due to the presence of SF. Fibre crack-bridging activity of SF, which improved the Q1 and Q2 of the D-PF-SF and D-SF-PF specimens, substantially impacted the mechanism of crack stabilization. This process was essential in enhancing impact strength by improving debonding, sliding, and drawing out of the fibre and delaying crack formation. Consequently, the impact strength of two-layered FTSFC specimens was lower than single-layered S-SF specimens. This could be due to more SF being distributed in the entire cross-section in the one-layered concrete specimen, while the two-layered FTSFC specimen contained SF in only one layer.

#### 4.2.3. Effects of Three-Layer FTSFC

**Figure 10:**
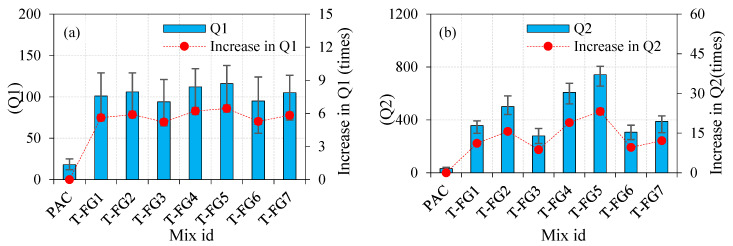
The effect of the triple-layered specimen on impact strength (**a**) Q1 (**b**) Q2.

The Q1 and Q2 values for the three-layered FTSFC specimens comprising mono- and hybrid SF and PF in different combinations are shown in Figure 10a,b. All FTSFC specimens showed an inherent increase in Q1 and Q2 when compared to the PAC specimen.

- The recorded Q1 and Q2 values for the T-FG1 specimen were 101 and 357, respectively. When compared to the PAC specimen, the observed values improved by 5.61 and 11.16 times, respectively.
- For the T-FG2 specimen, the recorded values for Q1 and Q2 were 106 and 501, respectively. The observed values were increased by 5.88 and 15.65 times, respectively, compared to the PAC specimen.
- The recorded Q1 and Q2 values for the T-FG3 specimen were 94 and 279, respectively, and these values were increased by 5.22 and 8.71 times, respectively.
- The Q1 and Q2 values for the T-FG4 specimen were 112 and 608, respectively. These values tended to increase by about 6.22 and 19 times, respectively.
- The recorded Q1 and Q2 values for the T-FG5 specimen under the optimal circumstances were 116 and 742, respectively. The recorded values increased by 6.44 and 23.18 times, respectively.
- The Q1 and Q2 values for the T-FG6 specimen were 95 and 307, respectively. The recorded values were increased by 5.27 and 9.59 times, respectively.
- For the T-FG7 specimen, values of 105 and 389 were recorded for Q1 and Q2, respectively. The recorded values were increased by 5.83 and 12.15 times, respectively.

All FTSFC specimens exhibited an excellent impact resistance compared to the PAC specimens. The highest Q1 and Q2 values were recorded for the T-FG5 specimen. This was due to the higher dosage of SF provided in the top and bottom layers, which increased the tensile strength of these layers. As a result of the compressive waves reflected produced by the falling mass, these layers could resist a more significant number of tensile waves formed at the specimen’s top surface. The ductile FTSFC specimen was designed to withstand massive tensile-stress waves mainly in the upper and bottom layers while also decreasing the energy wave via microcracking. Using a higher dosage of SF and PF in the top and bottom layers led to enhanced tensile strength and provided an additional impact-energy absorption mechanism owing to membrane action, which distributed impact stress over a larger area [81]. The performance of SF was better in all fibre schemes of three-layered FTSFC than that of PF. The composite element was taken into account by the created FTSFC layers. The concept behind FTSFC was to generate a steady development in finely structured materials that could meet the performance criteria of structural components. In this regard, FTSFC identified three interfacial transition zones. Due to the various dosages of fibres added into each layer of concrete, it had distinct characteristics. Through the interfacial transition zones, the transfer of shear stress occurred. Several layers may have reduced shear-stress transmission in the interfacial transition zone, leading to better composite action. All three layers worked together up to the final limit state, implying a strong connection between them. At the point of impact on the surface, a tough layer was created with a greater dosage of fibres. The findings indicated that the various SF and PF dosages used in the FTSFC increased the impact-energy absorption capability more substantially than the PAC specimen. The T-FG4 specimen exhibited the second-best impact-strength performance, with a hybrid combination of 2.8% SF + PF in both the top and bottom layers, and 1.6% SF + PF in the intermediate layer. It is worth noting that functionally graded concrete absorbs more impact energy under a falling mass impact than thoroughly reinforced cross-sections with the same quantity of fibres [26,81]. Increased fibre dosage resulted in better tensile properties in both the top and bottom layers, which led to increased impact strength.

#### 4.2.4. Impact Ductility Index (IDI) of FTSFC

**Figure 11:**
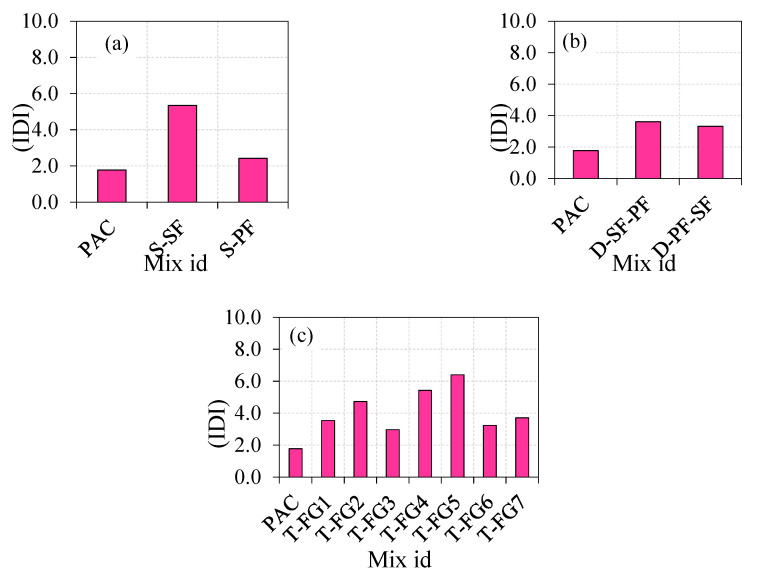
The impact ductility index (**a**) Single layer (**b**) Double layer (**c**) Triple layer.

The IDI was defined by the ratio of Q2 to Q1, and IDI values for all mixtures are shown in Figure 11. It can be seen in Figure 10 that the PAC specimen’s IDI value was 1.8, indicating an insufficient resistance to postcracking. The PAC specimens were quickly fractured into two or three pieces after the cracking. The IDI values of S-SF and S-PF were 5.3 and 2.4, respectively, indicating that the specimen that contained SF instead of PF had good postcrack resistance. However, the postcrack resistance of the three-layered FTSFC ranged from 3.0 to 6.4, which indicated an excellent postcrack resistance. During the crack initiation, fibres restricted the crack propagation by preventing the crack tip from opening within the concrete, thereby rejecting its brittleness and delaying crack growth [82]. The increased fibre dosage in FTSFC led to achieving a failure in a ductile manner. Higher IDI values indicate excellent ductile behaviour, and lower IDI values indicate brittle behaviour.

#### 4.2.5. Failure Pattern

**Figure 12:**
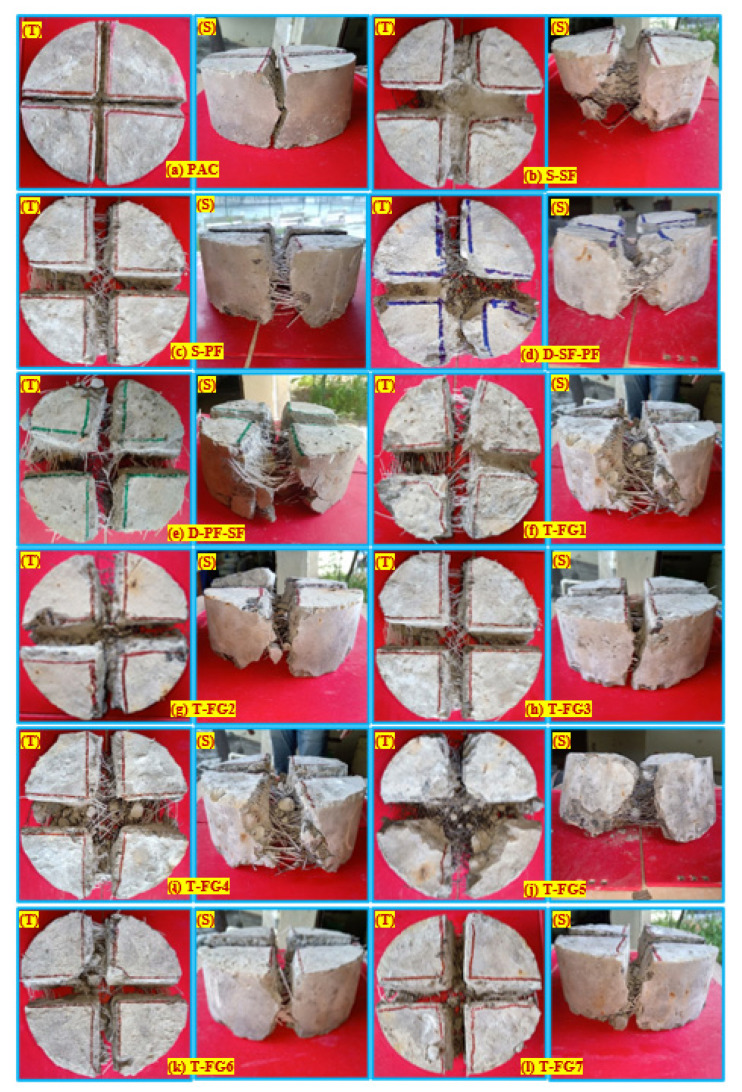
The failure pattern of the specimens under impact loading (**a**) PAC (**b**) S-SF (**c**) S-PF (**d**) D-SF-PF (**e**) D-PF-SF (**f**) T-FG1 (**g**) T-FG2 (**h**) T-FG3 (**i**) T-FG4 (**j**) T-FG5 (**k**) T-FG6 (**l**) T-FG7.

The failure of the FTSFC at the top and side face under impact loading is shown in Figure 12. The nonfibrous reference specimens were split into two pieces, displaying a brittle failure [26,69]. As illustrated in Figure 12a, the fracture propagated via the tip of a notch and approached the bottom surface of the specimen after some impacts for the cross-notched specimen. Figure 12b–l illustrate the behaviour of single-, double-, and three-layered FTSFC specimens that exhibited a ductile failure. After adding fibres to the concrete, the stress-transmission capacity across formed fractures was substantially enhanced. Improved bridging action of fibres on both sides of the cracked region prevented their growth. As a result of the increased energy-absorption capacity, the concrete achieved a higher impact-resistance capability [16]. The specimen’s top surface could withstand a greater impact number before breaking, as shown in Figure 12b,d. We noticed that the notched specimens cracked in a more controlled manner. As the frequency of impacts increased, the crack propagated along the depth of the notches, as shown in Figure 12a–l. The specimens with a cross notch produced fractures on both sides of the specimens, as well as the projection of the notch when they reached their cracking capacity. This failure pattern can be predicted due to the impact of notches and distributors of load, which can distribute stress along a cross-notch line and, as a result, regulate the fracture path. It was shown that this pattern of failure was consistent with previous studies [54,69]. This modification’s objective was to control the crack route, rather than to allow cracks to be distributed randomly.

#### 4.2.6. Orientation of Fibres in FTSFC

**Figure 13:**
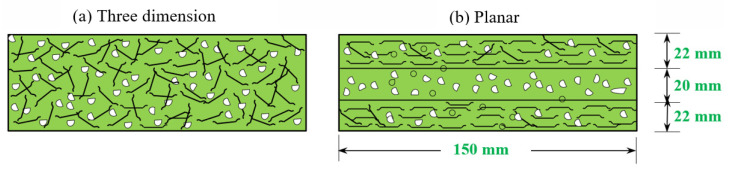
Orientation of fibres: (**a**) 3D; (**b**) planar.

The orientation of the fibres can affect the impact resistance of the composite [83,84,85]. All FTSFC specimens had a planar orientation of fibres in the majority of cases. Mastali et al. [81] reported that functionally graded fibrous concrete curved slabs with planar fibre alignment demonstrated greater impact strength than curved concrete slabs with a three-dimensional fibre orientation. Figure 13 depicts the appearance of the fibre arrangement in PAFC and FTSFC [2,26,80]. All T-FG series specimens featured planar-aligned fibre, while the S-SF and S-PF specimens exhibited a three-dimensional fibre orientation. The impact strength of three-layered T-FG5 specimens with planar fibre alignment was greater than that of the S-SF specimen with three-dimensional fibre orientation. Regardless of fibre orientation, the crack route in the specimens was mostly parallel to the direction of the cross-line notch [86,87]. Planar-oriented fibres perpendicular to the loading direction had greater resistance to cracking than the three-dimensional fibre arrangement. Thus, three layers of FTSFC specimens comprising planar-aligned fibres led to an improvement in their impact strength.

#### 4.2.7. Failure Mechanism of FTSFC Specimens

**Figure 14:**
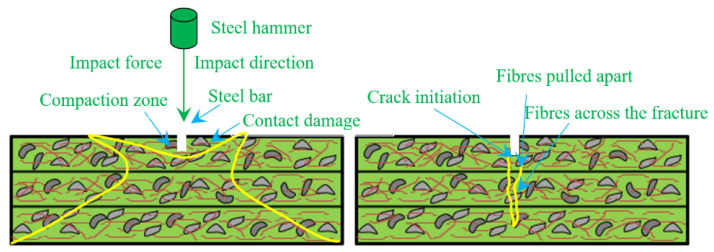
Failure mechanism.

Contact damage, failure of the matrix, failure of fibres, and additional fibres resulting from damage were seen in the specimens due to loading/delamination. These damaging effects occurred quickly, and it was thus difficult to explain their correct order. Figure 14 shows the process of damage resulting in the above-stated effects, including localized damages at the point of contact between the steel load transmission rod and the specimen. Transverse shear stress/strain led to delamination of the inner structure [88]. Tensile-wave transfer upon impact caused the matrix and fibres to debond into the surrounding areas, produced by tensile and compressive bending on the bottom and top surfaces, respectively. Delamination of fibres is an essential characteristic of failure, and affects the integrity of the composite matrix. In addition, these fibres lost more energy during secondary fracture formation, which is extremely difficult to identify during service. When a first fracture was formed, significant kinetic energy was transmitted into the fibres, limiting cracks and energy diffusion to neighboring areas.

#### 4.2.8. Comparison of ACI and Modified-Method Impact Results

**Figure 15:**
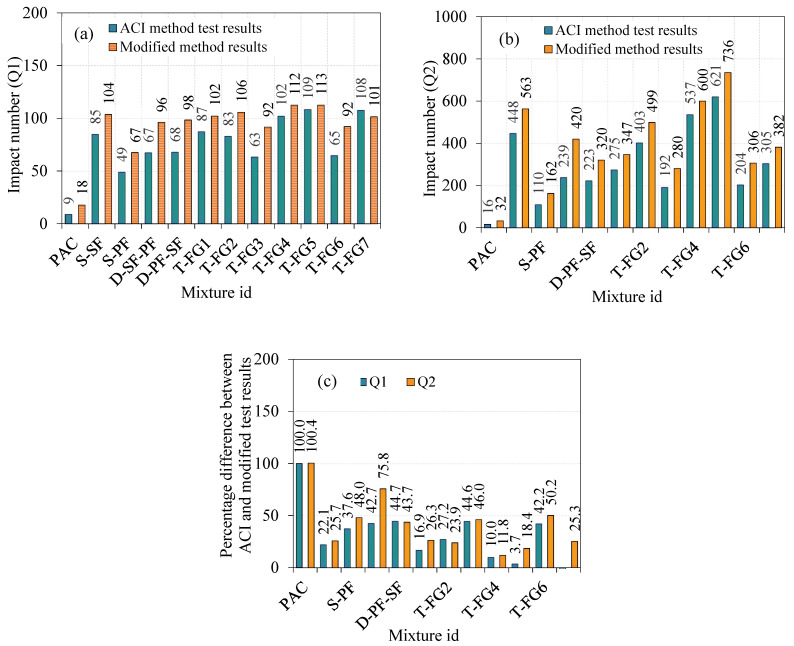
Comparison of the ACI and modified-method impact results (**a**) Q1 (**b**) Q2 (**c**) Percentage difference in Q1 and Q2.

Figure 15 shows a comparison of the modified test findings from this research with the ACI method testing results from a previous study [31] for the same mixes with identical fibre doses and schemes. As illustrated in Figure 15a,b, the modified impact technique produced substantially better impact test results for Q1 and Q2 than the ACI method. This pattern was consistent across all combinations, regardless of the concrete layer. As shown in Figure 15c, the percentage variation between the ACI and modified impact test results varied from −6 to 100% for Q1 and 11.8 to 100.4% for Q2. The specimens were exposed to a single point of impact, which applied a localized impact on a small specimen, resulting in lower Q1 and Q2 results for the ACI test technique. In the area of the aggregate, soft material and fibres, this single point impact took place in a compact cylindrical specimen, resulting in the concentration of impact energy in a limited area. This phenomenon caused a specimen to fracture and spread in a radial direction, resulting in a rapid collapse. On the other hand, the modified impact test yielded greater impact strength values for all FTSFC specimens. This occurred due to the impact load being dispersed over a wider area by horizontally placing the knife-edge cross-notch steel bar on the top specimen’s surface and repeatedly exposing it to the drop-weight impact. Consequently, the new test technique, single-point impact stress on a soft or hard region or the fibres, was eliminated. As a result of applying the line impact force that was dispersed over a large region, the specimens did not fail quickly.

#### 4.2.9. Comparison of the Coefficient of Variance (COV) Calculated from the ACI and Modified-Method Impact Test Results

**Figure 16:**
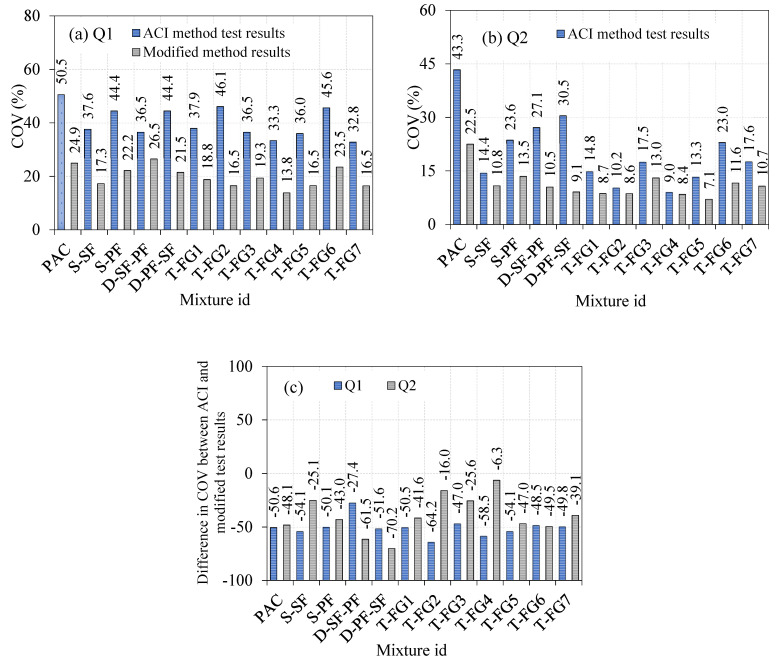
Comparison of COV with ACI and modified-method impact results (**a**) Q1 (**b**) Q2 (**c**) Percentage difference in Q1 and Q2.

The COV analysis showed the distribution of the impact-test findings. Increased and decreased COV values resulted in increased and decreased dispersion of impact-test findings, respectively. In general, a smaller COV was preferable in all cases because it reflected a more precise assessment. Figure 16 shows the COV calculated from the modified impact-test results versus the ACI method impact-test results from previous research [2]. The COV for Q1 and Q2 for the 12 combinations ranged from 32.8 to 50.5% and 9.0 to 43.3%, respectively. The prior sections explained the source of increased COV values from the ACI method impact test (Section 1.4). As seen in Table 1, several researchers revealed a significant COV from the drop-weight impact test. Drop-weight impact findings on various types of fibrous concrete clearly showed strong scattering in test results, which was consistent with previous research [45,53,55,56,58,66,67,68].

Similarly, modified impact-testing results revealed a reduced COV for all 12 mixes, ranging from 13.8 to 26.5% for Q1 and 7.1 to 22.5% for Q2. As shown in Figure 16c, the COV value estimated using the modified impact test was decreased, and varied between 27.4 and 64.2% in Q1 and 6.3 to 70.2% in Q2 compared to those for the ACI test method’s identical mixes. This occurrence clearly showed that the improved impact test provided reduced dispersion in findings by adding a cross-notch specimen that delivered impact utilizing a knife-edge steel bar on the top surface instead of a single-point impact. This study identified the causes of result dispersion, and proposed a strategy to mitigate these sources by laying the foundations for developing a new and improved impact-testing technique.

## 5. Conclusions

This study used a modified impact test for functionally graded two-stage fibrous concrete (FTSFC) against repeated low-velocity impacts. To minimise the dispersion of experimental results, a cross notch was created on the specimen, and a knife-edge steel bar was horizontally positioned to provide a parallel-line impact force rather than the usual single-point impact. Various mono- and hybrid schemes were derived from testing the impact resistance of single-, double-, and triple-layered concretes made of steel and polypropylene fibres. According to the thorough investigation, the following were the most important outcomes:

1. The S-SF specimen demonstrated the greatest compressive strength, with a 59.6% improvement over the reference specimen (PAC). The T-FG2 specimen, which had 2.8% SF in the top and bottom layers and 1.6% in the intermediate layer, had the second-greatest compressive strength increase at 54.4%. As a result, single-layered concrete outperformed three-layered FTSFC in compression tests. On the other hand, steel fibres contributed more to improving strength than PF, independent of the fibre scheme or the number of layers.
2. The reported Q1 increased by approximately 5.8 and 3.8 times for the S-SF and S-PF specimens, respectively. In comparison to PAC, the reported Q2 increased by about 17.5 and 5.2 times. For both Q1 and Q2, however, the effect of SF was greater than that of PF. This was caused by the introduction of fibres, which improved the matrix’s tensile capacity by providing high tensile-stress absorbance across fractures through crack spanning.
3. The T-FG group of specimens had higher Q1 and Q2 records than the PAC sample, which was expected due to the fibre-matrix reinforcing effect. The T-FG5 combination from this group had the most significant increases in Q1 and Q2, by about 6.4 and 23.2 times, respectively. This was due to the greater SF dose in the top and bottom layers, which were subjected to higher impact stresses due to the immediate contact with the supplied drop weight and the supportive base plate. Furthermore, the crimped and hooked-end structure of SF and its considerably greater tensile strength than PF contributed to its enhanced bond strength. The second-highest Q1 and Q2 values for three-layered FTSFC, recorded for the T-FG4 specimen, were 112 and 608, respectively. These values tended to increase by about 6.22 and 19 times, respectively. The third-highest recorded values were for the T-FG2 specimen, which had a Q1 and Q2 of 106 and 501, respectively. The observed values were increased by 5.88 and 15.65 times, respectively, compared to the PAC specimen.
4. The PAC had a ductility index value of 1.8, which indicated brittle failure. The ductility index value of all fibre specimens varied from 2.4 to 6.4, indicating a higher postcrack resistance. Moreover, a higher ductility was achieved by increasing fibre content in the top and bottom layers while reducing it in the intermediate layer.
5. Better controlled cracking behaviour was achieved by using notched specimens and load-transmitting plates. The cracks in notched specimens mainly started and spread along the edges of the notches, while the specimens examined per the ACI 544-2R method had numerous randomly dispersed cracks. This type of controlled activity would make it simpler to identify criteria for accepting or rejecting the findings of the specimens tested based upon their cracking pattern, reducing the dispersion of the results even further.
6. In comparison to the ACI technique, the modified-impact findings were considerably more significant. For Q1, the percentage difference between the ACI and modified impact test findings varied from −6 to 100%, while for Q2, the percentage difference ranged from 11.8 to 100.4%. Compared to the ACI test technique, the estimated COV values for all 12 mixes were reduced by 27.4 to 64.21% for Q1 and 6.3 to 70.16% for Q2. Consequently, the suggested impact-test modification can enhance the reliability of findings, is simple to perform, and contributes to emerging material technology.

## Figures and Tables

**Table 1:** The outline of the pilot experience of the ACI Committee, with and without fibres, for falling-mass impact outcomes on concrete.

Mix ID	Type of Composite	Dosage of Fibre (%)	Fibre Type	Sample Tested Per Mixture	Impact Failure Numbers	SD	COV (%)	Ref.
PC, CF1.5, CF3.0, CF5.0, HF1.5, HF3.0, HF5.0	Two stage fibrous concrete	1.5, 3.0, 5.0%	Crimped steel, hooked-end steel	15	84, 312, 737, 1209, 424, 918, 1378	25, 86, 113, 151, 64, 78, 122	30, 27, 15, 12, 15, 9, 9	[18]
GHPC, GHPSFRC	Green high-performance plain and FRC	0.5%	Steel	40	177, 240	81, 94	46, 39	[66]
M0, M1, M2, M3	Geopolymer fibre-reinforced concrete	1.6, 0.3, 0.3%	Steel, polypropylene, glass	5	14, 101, 32, 35	4.7, 20.3, 9.5, 11.7	33.5, 20.1, 30.1, 33.6	[45]
B1, B2	Fibre-reinforced concrete	3 kg/m^3^	Polypropylene fibre	20	84, 76	44, 37	52, 49	[58]
G1, G2	Fibre-reinforced concrete	2.5%	Steel	15	358, 417	207, 185	58, 44	[55]
SC30-0, SC30-0.5, SC30-0.75, SC30-1.0	Self-compacting fibre-reinforced concrete	0.5, 0.75, 1.0%	Steel	6	1.8, 7.3, 11.3, 17.2	0.8, 1.6, 1.6, 4.8	41.1, 22.3, 14.4, 27.9	[67]
HSFRC	High-strength fibre-reinforced concrete	1%	Hooked-end steel fibre	48	1896	802	42	[56]
M1	Fibre-reinforced concrete	2.5%	Steel	12	127	47	37	[68]
PC, CFRC, PRFC, SFRC	Fibre-reinforced concrete	0.15, 0.15, 0.5%	Cellulose fibre, polypropylene fibre, steel fibre	32	48, 118, 71, 228	28, 53, 36, 90	57, 45, 51, 39	[59]
NC, PP4, PP6, SF20, SF35	Fibre-reinforced concrete	4, 6, 20, 35 kg/m^3^	Polypropylene, steel	6	15, 33, 40, 52, 55	7, 7, 5, 27, 24	47, 21, 12, 52, 44	[53]

**Table 2:** The mixing composition of the composites.

Mix ID	Ratio of c/s	Ratio of w/c	Dosage of Fibre Used in the First Layer (%)		Dosage of Fibre Used in the Second Layer (%)		Dosage of Fibre Used in the Third Layer (%)		SP (%)
			SF	PF	SF	PF	SF	PF	
PAC	1.0	0.45	0						0.3
S-SF			SF (2.4)						0.4
S-PF			PF (2.4)						0.4
D-SF-PF			SF (2.4)			PF (2.4)			0.4
D-PF-SF			PF (2.4)			SF (2.4)			0.4
T-FG1			1.2	1.2	1.2	1.2	1.2	1.2	0.4
T-FG2			2.8	0	1.6	0	2.8	0	0.4
T-FG3			0	2.8	0	1.6	0	2.8	0.4
T-FG4			1.4	1.4	0.8	0.8	1.4	1.4	0.4
T-FG5			3.6	0	0	0	3.6	0	0.4
T-FG6			0	3.6	0	0	0	3.6	0.4
T-FG7			1.8	1.8	0	0	1.8	1.8	0.4

**Table 3:** Impact-test results.

Mix ID	PAC		S-SF		S-PF		D-SF-PF		D-PF-SF		T-FG1		T-FG2		T-FG3		T-FG4		T-FG5		T-FG6		T-FG7	
	Q1	Q2	Q1	Q2	Q1	Q2	Q1	Q2	Q1	Q2	Q1	Q2	Q1	Q2	Q1	Q2	Q1	Q2	Q1	Q2	Q1	Q2	Q1	Q2
1	12	18	79	456	48	122	68	274	71	280	71	299	75	441	65	222	85	521	85	656	56	251	72	303
2	12	22	81	486	49	129	69	281	73	284	76	305	79	449	69	235	88	535	88	661	61	258	78	315
3	13	25	85	491	51	136	71	298	77	288	78	315	84	459	72	241	93	546	90	672	66	261	83	332
4	14	27	89	502	54	141	73	301	81	291	84	319	90	464	76	249	99	551	91	685	74	273	88	353
5	15	29	93	532	56	153	76	311	82	296	88	327	96	471	79	256	104	562	97	701	80	285	92	361
6	15	30	96	542	59	158	78	322	83	302	95	331	101	479	84	263	109	575	105	716	86	293	94	372
7	15	31	99	555	61	165	83	340	88	312	99	343	107	486	88	271	114	584	110	730	88	299	99	384
8	17	33	102	569	65	168	87	345	94	321	104	347	109	491	92	277	116	601	117	741	93	308	103	394
9	18	35	109	587	68	170	94	355	101	329	111	356	112	500	91	286	119	615	120	751	97	317	106	401
10	19	37	113	595	74	175	101	360	108	334	114	361	115	505	98	294	121	632	124	763	101	321	109	409
11	20	38	119	601	79	178	121	374	114	345	116	368	119	515	104	305	123	638	128	777	108	329	112	414
12	22	39	124	612	82	180	126	384	119	351	120	374	121	528	109	315	125	646	130	782	112	337	116	415
13	23	40	128	621	86	184	130	394	125	353	122	381	123	543	112	326	127	656	131	794	116	341	121	419
14	24	41	134	644	89	188	131	399	129	359	124	385	125	575	115	331	130	662	134	802	119	356	122	424
15	25	42	138	656	90	190	135	409	131	361	129	392	129	582	121	335	134	677	138	806	124	360	126	430
Mean	18	32	104	563	67	162	96	420	98	320	102	347	106	499	92	280	112	600	113	736	92	306	101	382
SD	4.4	7.3	17.9	60.8	14.9	21.9	25.5	43.9	21.2	29.1	19.2	30.0	17.4	43.0	17.7	36.5	15.5	50.6	18.6	51.9	21.6	35.4	16.7	40.9
COV %	24.9	22.5	17.3	10.8	22.2	13.5	26.5	10.5	21.5	9.1	18.8	8.7	16.5	8.6	19.3	13.0	13.8	8.4	16.5	7.1	23.5	11.6	16.5	10.7

## Data Availability

Not applicable.

## Abbreviations

FTSFC	Functionally graded two-stage fibrous concrete
ACI	American Concrete Institute
PET	Polyethylene terephthalate
FRP	Fibre-reinforced polymer
CFRP	Carbon-fibre-reinforced polymer
FRC	Fibre-reinforced concrete
TSFC	Two-stage fibrous concrete
FGC	Functionally graded concrete
RFDWI	Repetitive falling drop weight or mass impact
COV	Coefficient of variance
PF	Polypropylene fibre
SF	Steel fibre
ASTM	American Society for Testing and Materials
Q1	Impact number causing initial crack
Q2	Impact number causing final crack
IDI	Impact ductility index
